# Oligodendrocyte Cell Line OLP6 Successfully Differentiates on Decellularized Brain Tissue

**DOI:** 10.14789/jmj.JMJ23-0007-OA

**Published:** 2023-06-22

**Authors:** KANA KATO, HINATA NISHIMURA, YUJI SUZUKI, TAKAHASHI TANAKA, RYUSEI ABE, AURELIEN KEREVER, ERI ARIKAWA-HIRASAWA

**Affiliations:** 1 Research Institute for Diseases of Old Age, Juntendo University Graduate School of Medicine, Tokyo, Japan; 1 Research Institute for Diseases of Old Age, Juntendo University Graduate School of Medicine, Tokyo, Japan; 2 Department of Neurology, Juntendo University Graduate School of Medicine, Tokyo, Japan; 2 Department of Neurology, Juntendo University Graduate School of Medicine, Tokyo, Japan

**Keywords:** oligodendrocyte, cell culture, decellularized brain tissue

## Abstract

**Objectives:**

The mechanisms of mental and neurological diseases have been proposed to be related not only to disorders of the neurons but also to the environment surrounding neurons, such as glial cells and the extracellular matrix (ECM). The chondroitin sulfate (CS) chain, which comprises CS proteoglycans (CSPGs), is one of the major sulfated glycosaminoglycans in the brain. CSPGs play an important role in the development, aging, and pathological conditions of the central nervous system. In particular, CSPGs play critical roles in oligodendrocyte differentiation and cell activity. Conventional two-dimensional culture in a glass chamber hardly replicates the complexity of the ECM structure or mimics *in vivo* conditions. Therefore, to solve this issue, this study aimed to use a culture system with decellularized tissue as a scaffold of organized ECM, thereby enabling the observation of cell differentiation and interactions between cells and the surrounding ECM.

**Materials and Methods:**

We investigated the differentiation potential of the OLP6 cell line using decellularized brain tissue as the substrate.

**Results:**

We observed that OLP6 differentiated faster on decellularized brain tissues than on conventional 2D-coated surfaces. The relative mRNA expression levels of *CNP*, *PNP*, and *MBP* as well as CSPGs were increased under 3D culture conditions.

**Conclusions:**

Our study provides the first evidence of the advantages of cell culture on decellularized tissues for the investigation of oligodendrocyte differentiation and cell/ECM interactions.

## Introduction

In recent years, the pathological mechanisms of psychiatric and neurological disorders have been suggested to be related not only to neuronal defects but also to the environment surrounding neurons, such as glial cells, oligodendrocytes, and extracellular matrix (ECM). Oligodendrocytes, a glial cell type, form myelin sheaths in the central nervous system (CNS), and their main function is to induce saltatory conduction and increase the conduction rate of action potentials. In addition, oligodendrocytes that do not form myelin have been shown to contact neurons and play a variety of roles in neuronal signaling^1, 2)^.

Several oligodendrocyte cell lines have been established using human oligodendrocytes^3)^, rat oligodendrocytes^4)^, and mouse oligodendrocytes^5)^. In this study, we used the OLP6 cell line derived from adult rat oligodendrocytes^6)^. This immortalized oligodendrocyte cell line harbors the temperature-sensitive Simian virus 40 large T-antigen gene, which allows the differentiation of oligodendrocytes in a timely manner when the culture temperature is increased from 33°C to 39°C. In recent years, the importance of the interaction between oligodendrocytes and ECM has been highlighted, in addition to the relationship between oligodendrocytes and neurons, *in vivo*. For example, chondroitin sulfate proteoglycan (CSPG), one of the components of the ECM, is produced by oligodendrocyte precursor cells (OPCs), and their chondroitin sulfate glycosaminoglycans (CS-GAGs) have been reported to inhibit oligodendrocyte differentiation^7)^. Thus, the relationship between oligodendrocytes and the ECM is of research interest to clarify the pathology of mental and nervous disorders. CSPGs have also been shown to inhibit OPC process outgrowth and differentiation *in vitro*^8)^. Currently, a 2D culture method using dishes has been established for culturing oligodendrocytes, and ECM molecules, such as laminin, fibronectin, and Matrigel, have been used as surface coatings to study their effects on oligodendrocyte primary culture^9)^. However, such 2D cultures fail to replicate the complexity of the ECM structure that exists in living organisms; therefore, it is not an appropriate tool to reproduce living organisms three-dimensionally and to observe the relationship between oligodendrocytes and the ECM. In addition, it was recently proposed that ECM stiffness plays a critical role in OPC activity^10)^. Thus, the complexity of ECM composition and structure cannot be investigated in a 2D culture setup.

Decellularization of tissues to obtain ECM with native composition and mechanical properties has recently been used to study the role of the ECM in stem cell differentiation^11, 12)^. Therefore, the present study aimed to present a protocol for brain tissue decellularization, in which only cellular components are removed from the tissue, and establish a 3D culture system for oligodendrocytes using the decellularized brain tissue as a substrate to culture oligodendrocytes in a three-dimensional environment that is closer to the *in vivo* conditions than conventional artificial culture substrates.

## Materials and Methods

### Animals

Eight ten-week-old C57BL/6J male mice, purchased from Jackson Laboratory, were used to create the decellularized brain tissue. All animal protocols were approved by the Animal Care and Use Committee of Juntendo University.

### Cell line

The OLP6 cell line (Neuroscience 2005; 136: 115- 121) was obtained from the Riken Cell Bank (Cell No. RCB2864) and was grafted on the decellularized brain tissue.

### Decellularization

The procedure for mouse brain section decellularization was modified from that reported by De Waele et al. (2015). Mice were perfused with 25 mL of ice-cold phosphate-buffered saline (PBS) containing 1% heparin. The brains were dissected, and 500 µm-thick coronal sections of the brain (between bregma 1 and bregma -1.5 mm) were obtained using a vibratome (VT1200S; Leica, Weltzar, Germany). The sections were first placed in demineralized water (dH_2_O) for 10 min and then incubated in dH_2_O containing 4% sodium deoxycholate (Fujifilm Wako Pure Chemical Industries Ltd., Tokyo, Japan). After washing with PBS, the sections were placed in dH_2_O and 5% Triton X-100 (Nacalai Tesque Inc., Kyoto, Japan), followed by another wash with PBS. This incubation sequence was repeated three times. Finally, DNA was eliminated by incubating the sections with 40 kU/mL DNaseⅠ (D4263; Sigma-Aldrich, St. Louis, MO, USA) diluted in 1M NaCl during the last cycle. The decellularized brain tissues were stored at 4ºC in PBS containing 2% penicillin/streptomycin (cat. 15140122; Gibco^TM^, Thermo Fisher Scientific, Waltham, MA, USA) and 0.8% amphotericin B. All incubations were at room temperature in a sterile environment in 24-well plates placed on a rotary shaker (NA-301N; Nisshin Rika Co., Ltd., Tokyo, Japan) at 100 rpm.

### 2D and 3D Cultures

**Figure 1:**
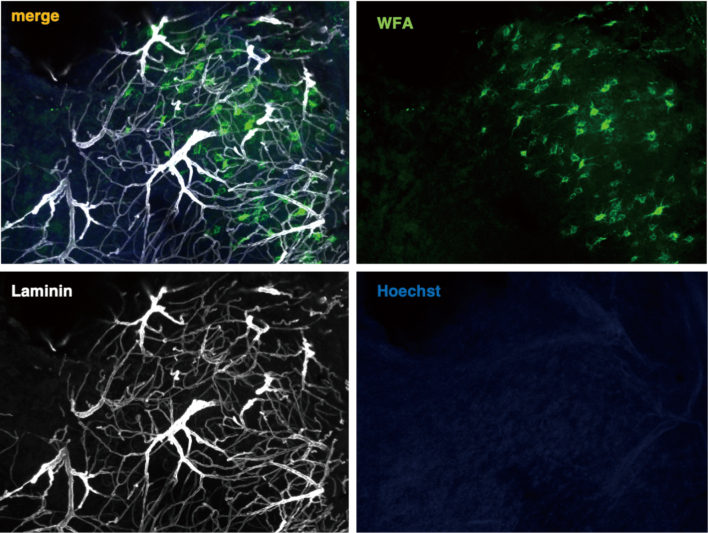
Confocal image of the decellularized brain tissue stained with laminin (white), Wisteria floribunda lectin (WFA) (green), and the nuclear marker Hoechst (blue).

OLP6 was maintained in culture buffer containing neurobasal medium (cat. 21103049; Gibco^TM^) supplemented with 5% fetal bovine serum, B27 supplement (cat. 17504044; Gibco^TM^), 0.5 mM glutamine (cat. 25030081, Gibco^TM^), 20 ng/mL epidermal growth factor (EGF; 100-15; Peprotech, Thermo Fisher Scientific), fibroblast growth factor (FGF)-2 (100-18C; Peprotech), and 1% penicillin/streptomycin (cat. 15140122; Gibco^TM^). The cell line was cultured under 5% CO_2_ at 33°C for proliferation and 39°C for differentiation. 2D and 3D cultures were performed according to the schedule shown in Figure 1. The cell proliferation period was 48 h. The medium was changed once every two days. In both the 2D and 3D samples, the time point for observation and analysis was set at 24 h from the start of differentiation.

#### 2D culture

OLP6 cells were cultured on 100 mm collagen I-coated dishes (cat. 4020-010; Iwaki, AGC Techno Glass Co. Ltd., Yoshida, Japan) at 33°C until confluence was achieved. For 2D culture observation, 500 µL of a medium at a density of 5 × 10^5^ cells was placed in each well of a Collagen I 8-well Culture Slide (cat. 354630; Corning BioCoat, Glendale, AZ, USA) and subjected to immunostaining.

#### Graft on the decellularized brain tissue for 3D culture

The decellularized brain tissue was rinsed in a chondroitinase buffer (50 mM Tris-HCl, 60 mM sodium acetate, 0.02% bovine serum albumin, pH = 8.0) containing 200 mU/mL chondroitinase ABC (C3667; Sigma-Aldrich) for 2 h at 37°C in to decompose and remove the chondroitin sulfate chains before the graft and then washed with PBS containing 2% penicillin/streptomycin and 0.8% amphotericin B. For the graft, 40 µL of a medium at a density of 2 × 10^5^ cells was poured onto half of the brain tissue. All animal experiments followed the Fundamental Guidelines for Proper Conduct of Animal Experiments and Related Activities in Academic Research Institutions under the jurisdiction of the Ministry of Education, Culture, Sports, Science and Technology (Notice No. 71, 2006) and were approved by the Committee for Animal Experimentation at Juntendo University (Approval No. 2022300).

### Immunohistochemistry

Samples were fixed in cold 4% paraformaldehyde (in PBS) for 20 min and washed with PBS. The samples were then placed in 0.5% Triton X-100/PBS solution for 15 min, followed by 15 min incubation in blocking solution (0.2% gelatin/PBS). Each antibody, diluted at a ratio of 1:400 in blocking solution (0.2% gelatin/PBS), was applied to the samples overnight at 4°C and then washed with PBS. The following primary antibodies and markers were used: laminin (rabbit polyclonal; Cat#L9393; 1:1000; Sigma-Aldrich), sox10 (rabbit monoclonal IgG; Cat# 11570; 1:1000; Abcam, Cambridge, UK), CNPase (mouse monoclonal IgG1; MAB326; 1:200; Merck KGaA, Darmstadt, Germany), Wisteria floribunda lectin (biotinylated; Cat#L1516; 1:800; Sigma-Aldric), Phalloidin-alexa488 (Cat# 11570; 1:400; Thermo Fisher Scientific). The sections were then rinsed, and secondary antibodies were applied for 60 min at 25°C. The secondary antibodies used were goat anti-rabbit Alexa Fluor-647, goat anti-mouse IgG1 Alexa Fluor-546, and streptavidin Alexa Fluor 488 (1:400 dilution in PBS; Thermo Fisher Scientific). After immunostaining, the cultured samples were incubated with nuclear dye (1 µL/mL in PBS; Hoechst, H3570; Thermo Fisher Scientific) for 20 min at 25°C. After extensive washing in PBS, the sections were mounted on a fluorogel with Tris buffer (Electron Microscopy Sciences, Hatfield, PA, USA). Imaging was performed within 24 h on an LSM780 microscope (Zeiss, Jena, Germany).

### Quantitative real-time PCR

RNA cell lysates were extracted using the Rneasy Mini Kit (Qiagen, Valencia, CA, USA). cDNA was synthesized using the RT2 First strand (#330404; Qiagen). Real-time PCR was performed using Fast SYBR Green Master Mix (#4385612; Applied Biosystems) on a Fast 7500 Real-Time Cycler (Applied Biosystems, Foster City, CA, USA). The gene GAPDH was used as an endogenous control. The primers used are listed in Table 1.

**Table 1:** List of primers used for quantitative real-time PCR

Gene	Forward primer	Reverse primer
*GAPDH*	ACTCTACCCACGGCAAGTTC	GATGGTGATGGGTTTCCCGT
*CNPase*	TTTGCCCGAAAAAGCCACACA	CACCGTGTCCTCATCTTGAAG
*MBP*	ACACAAGAACTACCCACTACGGC	CCAGCTAAATCTGCTGAGGGA
*PLP*	CCAGAATGTATGGTGTTCTCCC	GGCCCATGAGTTTAAGGACG
*Tenascin-R*	TCATCTCCATTACTGCTGAGAGG	AGTGCAAGTGGGAGATAGGG
*Phosphacan*	AACCATCCTTGGAAAACACG	CATTGGTGAGATTTATTTCCACTGT
*Brevican*	AGCAGAACCGCTTCAATGTC	TCAGAGGAAGCAGAGGGATG

### Statistical analysis

Statistical analysis was performed using PRISM statistical software (Prism 9, GraphPad Software, San Diego, CA, USA). Data are presented as mean ± standard error of the mean. Unpaired *t*-test or analysis of variance (ANOVA) with Tukey's multiple comparison test were performed.

## Results

### ECM structures are preserved after tissue decellularization

We first confirmed the removal of cell components and the condition of the ECM after decellularization. Hoechst staining of the decellularized tissue showed that all nuclear contents were cleared, while laminin immunostaining showed that the vascular basement membrane remained intact. In addition, Wisteria floribunda lectin staining showed that even fine ECM structures, such as perineuronal nets, were intact after the decellularization process (Figure 1).

### Differentiation of OLP6 in 2D and 3D cultures

**Figure 2:**
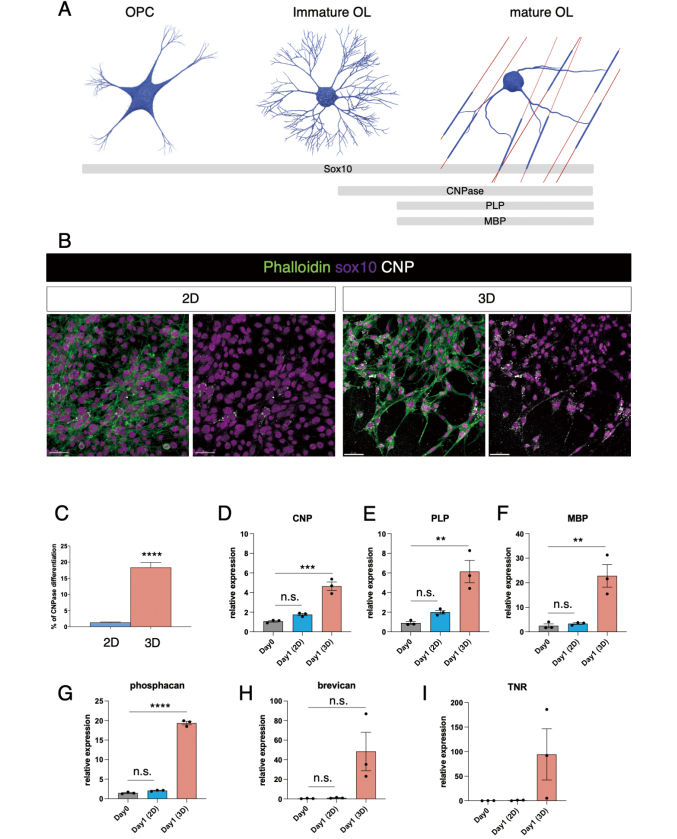
**A**. Schematic depiction of oligodendrocyte lineage and associated markers. **B**. Confocal images of OLP6 under 2D and 3D culture conditions after a 24-h differentiation period. **C**. Bar graph displays the ratio of sox10-positive cells that also expressed CNPase after 24 h of differentiation under 2D and 3D culture conditions. **D-F**. Bar graphs display the relative expression of oligodendrocyte differentiation markers on day 0 and after 24 h of differentiation under 2D and 3D culture conditions. (*** p = 0.0002; ** p = 0.0035 in E and 0.0045 in F, ANOVA with Tukey's multiple comparison test). **G-I**. Bar graphs display the relative expression of chondroitin sulfate proteoglycans *phosphacan*, *brevican*, and *tenascin-R* on day 0 and after 24 h of differentiation under 2D and 3D culture conditions. (**** p < 0.0001, ANOVA with Tukey's multiple comparison test).

To compare the differentiation of oligodendrocytes in 2D and 3D cultures, we cultivated OLP6 cells under differentiation conditions for 24 h (39°C, with serum, EGF, and FGF-2). A significantly higher proportion of sox10-positive cells also expressed the mature oligodendrocyte marker CNPase in the 3D culture (1.325 ± 0.1702% in 2D; 18.37 ± 0.1568% in 3D; p < 0.0001, unpaired *t*-test; Figure 2A-C). In addition, the RNA expression levels of the mature oligodendrocyte markers *CNP*, *PLP*, and *MBP*, were not significantly increased after differentiation for 24 h under 2D conditions (Figure 2D-F). However, the relative expression levels of *CNP*, *PNP*, and *MBP* on day 1 under 3D culture conditions increased by 3.5-fold (adjusted p = 0.0002, ANOVA with Tukey's multiple comparison tests; Figure 2D), 5.2-fold (adjusted p = 0.0002, ANOVA with Tukey's multiple comparison tests; Figure 2E), and 13-fold (adjusted p = 0.0045, ANOVA with Tukey's multiple comparison tests; Figure 2F), respectively, compared those on day 0. We also observed that the expression levels of CSPGs dramatically increased under 3D conditions. The relative *phosphacan* expression level on day 1 in the 3D condition increased by 17-fold compared to that on day 0 (adjusted p < 0.0001, ANOVA with Tukey's multiple comparison tests; Figure 2G). Additionally, the relative *brevican* and *tenascin-R* expression levels were high on day 1 under 3D conditions but barely detectable on day 0 or day 1 under 2D conditions (Figure 2H, I).

## Discussion

Oligodendrocyte differentiation markers, such as CNP and MBP, have been previously used to characterize immature and mature oligodendrocytes during OLP6 differentiation^6)^. In this study, we found that mature oligodendrocyte markers were more rapidly expressed when OLP6 cells were allowed to differentiate on decellularized tissues rather than on commonly used coated surfaces. The production of CSPGs, such as tenascin-R, by oligodendrocytes has been implicated in neural cell recognition^13)^. In addition, CSPGs, such as versican, are produced by oligodendrocyte lineage cells in response to an injury^14)^. In this study, we report that the ECM expression profile of different CSPGs was significantly increased when OLP6 was differentiated on decellularized tissues. Although decellularized brain tissue has been previously used as a scaffold for the long-term growth of undifferentiated neural stem cells^11)^, to the best of our knowledge, the present study is the first to report oligodendrocyte cell line differentiation on decellularized brain tissues.

This preliminary study focused on the onset of OPC differentiation into mature oligodendrocytes. However, further studies in which OLP6 can be differentiated for a longer period are necessary to fully understand the benefit of growing them on decellularized tissue. Nonetheless, this study highlights a new model of 3D *in vitro* oligodendrocyte culture that contains ECM with all of its chemical and mechanical complexities. Our study provides important insights into the development of models to investigate the OPC/ECM interaction.

## Funding

A Grant-in-Aid was provided by JSPS KAKENHI, under grant numbers 20K08089 to AK and 22K07543 to EAH.

## Author contributions

KK, HN, RA, YY, and TT designed the study and wrote the manuscript, and AK and EAH supervised the work and revised the manuscript. All authors read and approved the final manuscript.

## Conflicts of interest statement

The authors declare that there are no conflicts of interest. All experiments were conducted in compliance with the ARRIVE guidelines.
